# Synthesis of tenascin and laminin beta2 chain in human bronchial epithelial cells is enhanced by cysteinyl leukotrienes via CysLT_1 _receptor

**DOI:** 10.1186/1465-9921-9-44

**Published:** 2008-05-26

**Authors:** Siiri Altraja, Martin Kadai, Erki Rekker, Alan Altraja

**Affiliations:** 1 Institute of General & Molecular Pathology, University of Tartu, Tartu, Estonia; 2 Estonian Biocentre, Tartu, Estonia; 3 Institute of Molecular & Cell Biology, University of Tartu, Tartu, Estonia; 4 Department of Pulmonary Medicine, University of Tartu, Tartu, Estonia

## Abstract

**Background:**

Cysteinyl leukotrienes (CysLTs) are key mediators of asthma, but their role in the genesis of airway remodeling is insufficiently understood. Recent evidence suggests that increased expression of tenascin (Tn) and laminin (Ln) β2 chain is indicative of the remodeling activity in asthma, but represents also an example of deposition of extracellular matrix, which affects the airway wall compliance. We tested the hypothesis that CysLTs affect production of Tn and Ln β2 chain by human bronchial epithelial cells and elucidated, which of the CysLT receptors, CysLT_1 _or CysLT_2_, mediate this effect.

**Methods:**

Cultured BEAS-2B human bronchial epithelial cells were stimulated with leukotriene D_4 _(LTD_4_) and E_4 _(LTE_4_) and evaluated by immunocytochemistry, Western blotting, flow cytometry, and RT-PCR. CysLT receptors were differentially blocked with use of montelukast or BAY u9773.

**Results:**

LTD_4 _and LTE_4 _significantly augmented the expression of Tn, whereas LTD_4_, distinctly from LTE_4_, was able to increase also the Ln β2 chain. Although the expression of CysLT_2 _prevailed over that of CysLT_1_, the up-regulation of Tn and Ln β2 chain by CysLTs was completely blocked by the CysLT_1_-selective antagonist montelukast with no difference between montelukast and the dual antagonist BAY u9773 for the inhibitory capacity.

**Conclusion:**

These findings suggest that the CysLT-induced up-regulation of Tn and Ln β2 chain, an important epithelium-linked aspect of airway remodeling, is mediated predominantly by the CysLT_1 _receptor. The results provide a novel aspect to support the use of CysLT_1 _receptor antagonists in the anti-remodeling treatment of asthma.

## Background

Airway remodeling is a fundamental abnormality in the pathobiology of inflammatory airway diseases like bronchial asthma and chronic obstructive pulmonary disease (COPD) [1,2]. This remodeling is irreversible in its many aspects including airway fibrosis and deposition of new extracellular matrix (ECM) components into the airway wall [2]. These events lead to a thickened airway wall with markedly reduced airway caliber resulting in progressive development of fixed airflow limitation [2-4]. In asthmatics, increased deposition of collagen types I and III, but also tenascin (Tn) and laminin (Ln) chains α2 and β2 beneath the airway epithelium have been described [5-7], whereas the subepithelial Tn deposition is severity-dependent and can be reduced by anti-inflammatory treatment [7]. In bronchial epithelial cells *in vitro*, Tn can be up-regulated by cytokines like transforming growth factor-β (TGF-β) [8] and tumor necrosis factor-α (TNF-α) [9], which are also operative in asthma. Judged by rapid turnover of Tn in the bronchial mucosa [10], the up-regulation of Tn in asthma may refer to its role in inflammation and immediate repair. However, in addition to serving as a marker of ongoing inflammation, the deposition of Tn can contribute to increased airway narrowing and decreased airway wall compliance, as does the deposition of other ECM components.

Experimental data suggest that cysteinyl leukotrienes (CysLTs), known mainly as effector mediators in asthma, induce a broad spectrum of pathophysiological responses [11]. CysLTs contribute to airway remodeling not only through their diverse roles in inflammation, but also by their direct effects on lung mesenchymal cells [12-15]. CysLTs mediate their diverse effects through at least two G-protein coupled receptors, CysLT_1 _and CysLT_2_. Both receptors have been described in a variety of human cells, including inflammatory cells like monocytes/macrophages, mast cells and eosinophils [16,17], expressed predominantly on the outer plasma membrane. Recently, also an agonist-regulated localization of the CysLT_1 _in the outer nuclear membrane was demonstrated in certain cells [18,19].

Specific antagonists for CysLT_1_, such as montelukast and zafirlukast, have been developed as active controller medicines for asthma [20], whereas a dual antagonist, BAY u9773, is available for experimental use [21]. Apart from reduction of airway subepithelial fibrosis by blockade of the CysLT_1 _receptor with montelukast in a murine model [22] and attenuation of airway myofibroblasts by montelukast in human asthmatics following allergen challenge, the knowledge about the effects of antileukotrienes on remodeling of the airway ECM in humans is overall very limited [11,15]. Since a failure in the injury-repair cycle of the inflamed bronchial epithelium seems to play a major role in the remodeling via development of abnormally activated epithelial-mesenchymal interactions [3,11,23,24], a study was undertaken on human bronchial epithelial cells to investigate whether CysLTs have a direct effect on the synthesis of Tn and Ln β2 chain, proteins thought both to reflect the activity of the ongoing inflammatory remodeling and to affect airway compliance in asthma. In this study, we also checked the differential expression of both CysLT receptors on these cells and showed that although CysLT_2 _is significantly more abundant in human bronchial epithelium, the enhanced expression of the ECM proteins is mediated singly by CysLT_1_.

## Materials and methods

### Cell culture

Normal human bronchial epithelial cells (line BEAS-2B) were obtained from the American Type Culture Collection (Manassas, VA, USA). The cells were cultured at 37°C in a humidified atmosphere of 5% CO_2 _using serum-free epithelial growth medium (BEGM; Cambrex, Walkersville, MD, USA) supplemented with Bullet Kit (Cambrex) to contain 0.5 ng/ml human recombinant epidermal growth factor, 50 μg/ml bovine pituitary extract, 0.5 μg/ml hydrocortisone, 5 μg/ml insulin, 10 μg/ml transferrin, 0.5 μg/ml epinephrine, 0.1 ng/ml retinoic acid, 6.5 ng/ml triiodothyronine, 50 μg/ml gentamicin and 50 ng/ml amphotericin B. After reaching 50% subconfluency, the cells were challenged with 0 to 100 nM concentrations of either LTD_4_, the most potent or LTE_4_, the weakest though the most stable of the CysLTs (both from Cayman Chemical, Ann Arbor, MI, USA) during 24, 48, and 72 hours. TGF-β _1 _(1 ng/ml) (Chemicon, Hampshire, UK), known to stimulate Tn expression in BEAS-2B cells [8], served as a positive control. Montelukast sodium (1 μM, Merck-Frosst, Pointe Clarie, Quebec, Canada) or BAY u9773 (1 μM, Cayman Chemical) was added to the cell culture for 30 min before challenging with LTD_4 _or LTE_4 _to block the CysLT effect mediated via CysLT_1 _or both CysLT receptors, respectively. In settings, where LTD_4_-stimulation was involved, parallel experiments were performed with the presence of 5 mM L-cysteine (Sigma, St. Louis, MO, USA.) in the media to inhibit catabolism of LTD_4 _into LTE_4_.

### Antibodies

Mouse monoclonal antibody (clone 100EB2), which recognizes the fourth and fifth fibronectin-like repeats in Tn-C [25] and reacts with two different Tn polypeptides, was kindly donated by Prof. I. Virtanen (University of Helsinki, Finland). Mouse monoclonal antibody (clone C4) against β2 chain of human Ln was obtained from the Developmental Studies Hybridoma Bank (University of Iowa, Iowa City, IA, USA). Rabbit polyclonal antibodies against human CysLT_1 _and CysLT_2 _were from Cayman Chemical. Horseradish peroxidase (HRP)-conjugated rabbit anti-mouse and swine anti-rabbit antibodies, fluorescein isothiocyanate (FITC)-conjugated rabbit anti-mouse and swine anti-rabbit antibodies, as well as appropriate mouse and rabbit isotype control antibodies to provide negative controls were from Dako (Glostrup, Denmark).

### Western blotting

Confluent cells were washed with cold PBS and lysed in SDS sample buffer (62 mM Tris pH 6.8, 2% SDS, 0.1 M dithiotreitol, 10% glycerol and 0.05% bromophenol blue). Equal protein amounts (70 μg) were separated by 6.5% or 8% SDS/polyacrylamide gel electrophoresis and transferred onto polyvinylidene difluoride (PVDF) membranes (0.45 μm, Micron Separations Inc., Westborough, MA, USA), followed by blocking with 3% BSA in TBS/T (10 mM Tris-HCl pH 7.5, 0.1 M NaCl and 0.05% Tween-20) overnight at 4°C and probing for 1 h at room temperature with primary antibody against Tn, Ln β2 chain, CysLT_1_, or CysLT_2 _(1:50, 1:50, 1:250, and 1:250, respectively). Expression of CysLT_1 _and CysLT_2 _were analyzed in the presence or absence of appropriate specific receptor blocking peptides (both from Cayman Chemical, at 1:1 ratio to the respective antibody) with two different amounts of total BEAS-2B cell protein loaded (35 and 70 μg). After incubation, blots were washed twice for 10 min with TBS/T, followed by incubation with the appropriate HRP-conjugated secondary antibodies for 1 h at room temperature. The immunoreactivity was visualized with 3,3'-diaminobenzidine tetrahydrochloride (Sigma, St. Louis, MO, USA) reaction. To detect Tn and Ln β2 chain, the respective culture media were analyzed analogously.

### Flow cytometry

For flow cytometry studies, the BEAS-2B cells were washed with PBS, fixed in 2% paraformaldehyde in PBS for 15 min at room temperature and permeabilized in PBS containing 0.1% saponin. A total of 3.0 × 10^5 ^cells were resuspended in PBS supplemented with 3% BSA and 0.1% saponin for 30 min. All subsequent steps were performed in PBS containing saponin and BSA. The cells were incubated with the primary antibody against Tn, Ln β2 chain, CysLT_1_, or CysLT_2 _for 30 min, washed three times and incubated with the appropriate FITC-conjugated secondary antibody for 30 min. After washing, cells were resuspended in PBS and single-color immunofluorescence analysis of 15 000 cells was performed on a FACS Calibur flow cytometer using CELLQuest software (Becton Dickinson, San Jose, CA, USA). Negative controls were provided by substituting the primary antibody with an appropriate isotype control antibody.

### Immunocytochemistry

Cells grown on coverslips were fixed with 4% paraformaldehyde in PBS for 15 minutes and permeabilized with 0.1% Triton X-100 in PBS for 10 minutes. The fixed cells were blocked with 3% BSA in PBS prior to incubation for 1 h at room temperature with the primary antibodies against Tn, Ln β2 chain, CysLT_1_, or CysLT_2 _(1:10, 1:10, 1:50, and 1:50, respectively). The coverslips were washed three times with PBS and probed with appropriate HRP-conjugated secondary antibodies. The brown immunoperoxidase reaction was developed with diaminobenzidine, followed by hematoxylin counterstaining. The coverslips were mounted face down on microscope slides using a Permanent Mounting Media (Dako). The CysLT receptors were alternatively immunofluorescence-stained with FITC-conjugated swine anti-rabbit secondary antibody for 1 h at room temperature and mounted on slides using a fluorescence mounting medium (Dako). The cells were examined with an Olympus BX61 microscope (Olympus Optical Co., Ltd., Tokyo, Japan) and images were obtained using an Olympus DP70 digital camera and DP70-BSW software (version 01.02.). Negative controls were attained by substituting the primary antibody with an appropriate isotype control antibody.

### RNA isolation and RT-PCR

Total RNA was isolated from cultured BEAS-2B cells using the guanidinium-thiocyanate-phenol-chloroform extraction method [26]. Total RNA concentration was determined on NanoDrop ND-1000 spectrophotometer (NanoDrop Technologies, Inc., Wilmington, DE, USA) and purity was checked by the A_260_/A_280 _ratio (ratios between 1.9 and 2.1 were considered acceptable), in addition, an agarose gel was run to assess the quality. cDNA was synthesized from 5 μg RNA with RevertAid™ H Minus Moloney murine leukemia virus reverse transcriptase (Fermentas, Vilnius, Lithuania) using oligo(dT)_18 _priming according to manufacturer's instructions. The resulting cDNA samples were amplified by polymerase chain reaction (PCR) using a DNA thermal cycler (MJ Research, Watertown, MA, USA) and the following specific oligonucleotide primer pairs: for Tn, forward 5'-CAGCTCCACACTCCAGGTAC-3' and reverse 5'-CTTTCGCTGGGCTCTGAAGG-3' giving a PCR product of 448 bp, for Ln β2 chain, forward 5'-GCTCGGCAGTTGGATGCTCTC-3' and reverse 5'-GCCCGCTCATTTTCCTCATAG-3' giving a PCR product of 315 bp, for CysLT_1_, forward 5'-AGCCCCCACAAGACAATCAA-3' and reverse 5'-AGGAGAGGGTCAAAGCAACAA-3', providing a PCR product size of 358 bp, for CysLT_2_, forward 5'-GCAACCATCCATCTCCGTATC-3' and reverse 5'-CCAGGAAACGCACAACACTC-3', that gives a PCR product of 392 bp, and for β-actin, forward 5'-TCCCTGGAGAAGAGCTACGA-3' and reverse 5'-ATCTGCTGGAAGGTGGACAG-3', giving a PCR product of 362 bp. After an initial denaturation at 94°C for 3 min, PCR mixtures were amplified by 35 cycles consisting of 94°C for 25 s, 58°C for 30 s, 72°C for 30 s for Tn, by 30 cycles consisting of 94°C for 20 s, 57°C for 25 s, 72°C for 30 s for Ln β2 chain and 35 cycles consisting of 94°C for 10 s, 60°C for 20 s and 72°C for 30 s for the CysLT receptors, with a final extension at 72°C for 5 min. Amplified PCR products were separated by 2% agarose gel electrophoresis, visualized by ethidium bromide and photographed under ultraviolet transillumination together with the 100 bp DNA ladder marker (Fermentas). Omitting either the cDNA from the PCR reaction or reverse transcriptase from the cDNA synthesis reaction provided negative controls.

### Data analysis

The densitometric analysis of the Western blot and RT-PCR results was performed with use of the Image Pro Plus software (version 4.0, Media Cybernetics Inc., Silver Spring, MD, USA) and the results are expressed as means ± SEM. For statistical analyses, the SPSS statistical package (version 12.0) (Chicago, IL, USA) vas applied. Significance was determined by Kruskal-Wallis test with subsequent *post hoc *analysis between stimulated and control cells using Dunn's multiple comparison test. Two-tailed Mann-Whitney rank sum test was applied for comparison between data of two groups.

## Results

### Expression of tenascin

**Figure 1:**
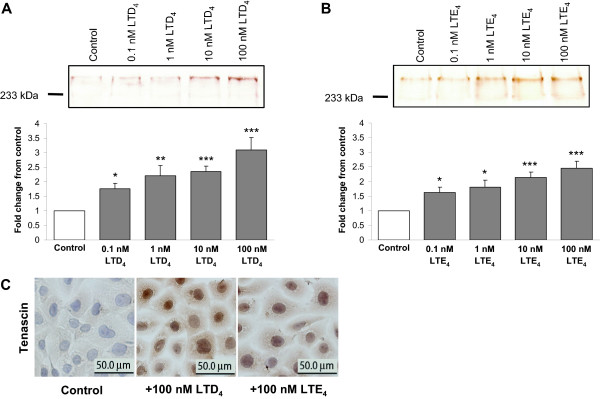
**Effect of LTD_4 _and LTE_4 _on the expression of tenascin (Tn) protein in BEAS-2B human bronchial epithelial cells**. A concentration-dependent increase in expression of Tn was detected by Western blot analysis in cell lysates after exposure of the cells to LTD_4 _(A) and to LTE_4 _(B) for 48 h. The data were quantified by densitometry and are expressed as fold increases above non-stimulated cells (mean ± SEM) (n = 12). C: Immunoperoxidase immunocytochemistry for Tn showing the basal expression of Tn and an increase by treatment with LTD_4 _and with LTE_4_. Images are reproduced from a single experiment, but are representative findings of multiple staining experiments. *p < 0.05, **p < 0.01, and ***p < 0.001 vs. non-stimulated cells.

**Figure 2:**
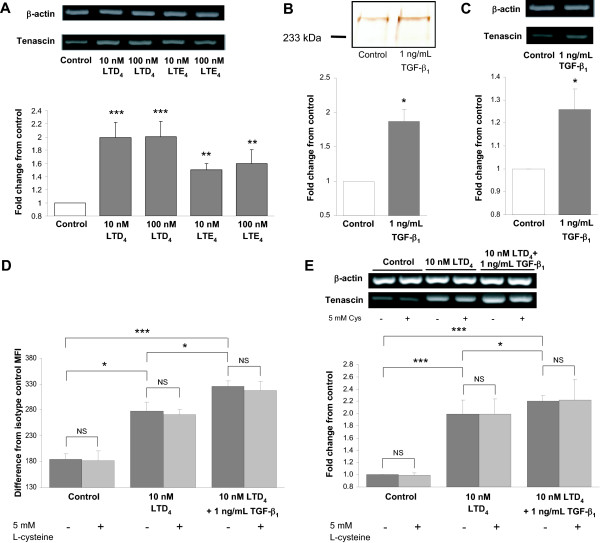
**Effect of LTD_4_, LTE_4_, and TGF-β_1 _on the expression of tenascin (Tn) in BEAS-2B human bronchial epithelial cells**. RT-PCR analysis detected increased expression of Tn mRNA in response to stimulation with LTD_4 _and LTE_4 _(A), TGF-β_1 _(C), and LTD_4 _combined with TGF-β_1 _(E) for 48 h. B: Increased expression of Tn was detected by Western blot analysis in cell lysates after stimulation with TGF-β_1 _(B) for 48 h. The data were quantified by densitometry and are expressed as fold increases above non-stimulated cells (mean ± SEM) (n = 12). D: Augmented expression of Tn protein was detected by flow cytometry in response to LTD_4 _combined with TGF-β_1_, compared with the effect of LTD_4 _alone. Difference from isotype control mean fluorescence intensity (MFI) is presented (mean ± SEM). Presence of 5 mM L-cysteine in the culture media did not affect the expression levels of Tn (D, E). *p < 0.05, **p < 0.01, and ***p < 0.001 vs. non-stimulated cells. NS – non-significant.

Immunoblot analysis of the non-stimulated control cells demonstrated two bands of Tn-immunoreactivity representing polypeptides of M_r _280,000 and M_r _230,000 (Figures 1A, B, and 2B). Exposure of the cells to both LTD_4 _and LTE_4 _for 48 h augmented the expression of the Tn polypeptide doublet, as well as Tn mRNA in a concentration-dependent manner (Figures 1A, B, and 2A). No significant time-dependence was seen, as analysis of the cells stimulated for 24 and 72 h gave similar results (data not shown). TGF-β_1 _also produced a significant increase in Tn at both protein (Figure 2B) and mRNA level (Figure 2C). No Tn was detectable in the culture media, regardless of the treatment setting.

Immunocytochemically, Tn-specific reactivity localized intracellularly in the BEAS-2B cells (Figure 1C) and, like that detected with immunoblot, it increased in response to both LTD_4 _and LTE_4_. Results from experiments combining 1 ng/ml TGF-beta and 10 nM LTD_4 _revealed an additive effect of both mediators on Tn expression. In particular, the increase in Tn expression represented approximately the sum of the effects achieved by both mediators alone, assessed by both flow cytometry (Figure 2D) and RT-PCR analysis (Figure 2E). Presence of 5 mM L-cysteine in the culture media did not affect the performance of LTD_4 _in relevant experimental series (Figures 2D and 2E).

### Expression of laminin β2 chain

**Figure 3:**
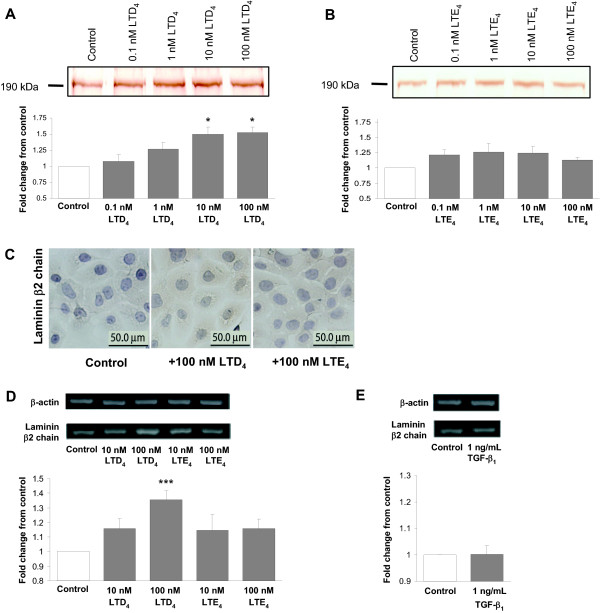
**Effect of LTD_4_, LTE_4 _and TGF-β_1 _on the expression of laminin (Ln) β2 chain in BEAS-2B human bronchial epithelial cells**. Western blot analysis revealed increased expression of Ln β2 chain in the cell culture medium in response to stimulation with LTD_4 _(A), but not with LTE_4 _(B) for 48 h. RT-PCR analysis detected increased expression of Ln β2 chain mRNA in response to 100 nM LTD_4 _(D), but no effect with either LTE_4 _(D) or TGF-β_1 _(E). The increase in response to LTD_4 _was concentration-dependent. The data were quantified by densitometry and are expressed as fold increases above non-stimulated cells (mean ± SEM) (n = 12). *p < 0.05 and ***p < 0.001 vs. non-stimulated cells. C: Immunoperoxidase immunocytochemistry for Ln β2 chain showing no definite expression of Ln β2 chain by non-stimulated control cells with a slight increase after treatment with both LTD_4 _and LTE_4_. Images are reproduced from a single experiment, but are representative findings of multiple staining experiments.

Immunoblot analysis revealed an insignificant increase in the expression of Ln β2 chain by the BEAS-2B cells from the basal level after stimulation with LTD_4 _and LTE_4 _for 48 h (data not shown). Immunocytochemistry showed a faint intracellular staining following stimulation with both CysLTs (Figure 3C). However, unlike that with Tn, Western blot analysis of the culture media for Ln β2 chain displayed already a detectable baseline level (Figures 3A and 3B). Media from cultures treated with 10 and 100 nM LTD_4 _(Figure 3A) but not with LTE_4 _(Figure 3B) showed a significant increase in Ln β2 chain, but no time-dependence was observed (data not shown). The expression of Ln β2 chain mRNA was significantly up-regulated in BEAS-2B cells by only 100 nM LTD_4 _(Figure 3D). TGF-β_1 _did not affect the expression of Ln β2 chain at either protein (data not shown) or mRNA (Figure 3E) level.

### Expression of CysLT_1 _and CysLT_2 _mRNA and protein

**Figure 4:**
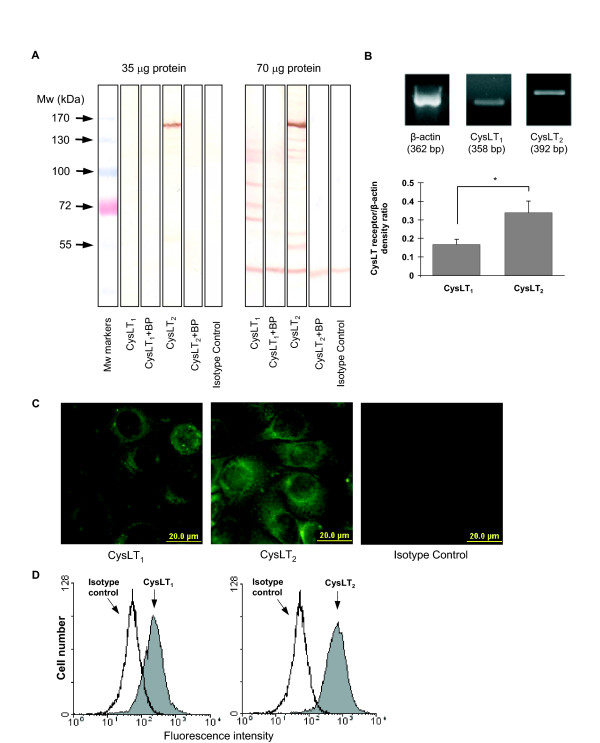
**Analysis of cysteinyl leukotriene receptors CysLT_1 _and CysLT_2 _in BEAS-2B human bronchial epithelial cells**. A: Western blot analysis of CysLT_1 _and CysLT_2 _protein in the presence or absence of specific blocking peptide (BP) with increasing amount of protein loaded. B: RT-PCR analysis of CysLT_1 _and CysLT_2 _mRNA. The graph represents densitometric results of CysLT_1 _and CysLT_2_, normalized to β-actin (mean ± SEM) (n = 9). *p < 0.05. C: Indirect FITC-immunofluorescence staining showing localization of cysteinyl leukotriene receptors in non-stimulated cells. D: Representative flow cytometric analysis of CysLT_1 _and CysLT_2 _on BEAS-2B cells. The open histograms correspond to cells labeled with isotype control antibody and the shaded ones represent cells labeled with the respective anti-CysLT antibodies.

For CysLT_1_, immunoreactive protein bands with molecular weights of approximately 66, 76, 88, 106, and 111 kDa appeared on Western blots, whereas CysLT_2 _appeared as 55-, 62-, 117-, 122-, 131-, and a major 148-kDa band (Figure 4A). All these bands were blocked with the respective blocking peptides. At lower protein load (35 μg), only a 148-kDa band for CysLT_2 _was visible. Immunocytochemistry also confirmed a positive reactivity for both CysLT_1 _and CysLT_2 _on BEAS-2B cells that consisted mainly of a bright cytoplasmic staining in all visualized cells. The staining pattern was similar for both receptors, however, immunoreactivity for the CysLT_2 _was stronger (Figure 4C). Flow cytometric analysis confirmed the presence of both CysLT_1 _and CysLT_2 _on BEAS-2B bronchial epithelial cells, as well as corroborated the more vigorous fluorescence for CysLT_2 _compared to that for CysLT_1 _(Figure 4D).

RT-PCR analysis revealed the amplified fragments for CysLT_1 _and CysLT_2 _that corresponded to the respective expected sizes of the transcripts (Figure 4B). The signal obtained for CysLT_2_, normalized to β-actin expression, was somewhat higher than that for the CysLT_1_.

### Effect of antileukotrienes on CysLT-induced up-regulation of ECM proteins

**Figure 5:**
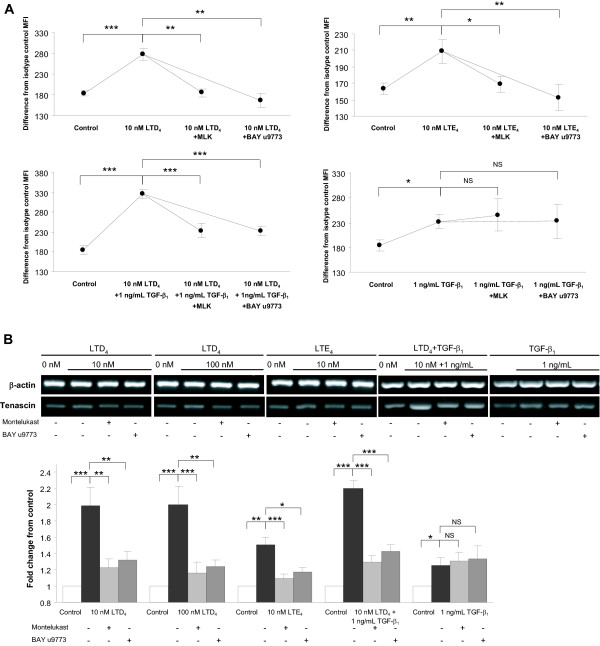
**Inhibition by antileukotrienes of tenascin (Tn) expression in cysteinyl leukotriene-stimulated BEAS-2B human bronchial epithelial cells**. A: Flow cytometric analysis showed full inhibition by both montelukast (MLK) and BAY u9773 of LTD_4_- or LTE_4_-induced increase in Tn protein expression to the levels not differing from that in non-stimulated cells. Difference from isotype control mean fluorescence intensity (MFI) is presented. B: RT-PCR analysis of Tn mRNA showing inhibition of the cysteinyl leukotriene-induced up-regulation by both MLK and BAY u9773. Partial inhibition of Tn expression induced by combined LTD_4 _and TGF-β_1 _down to the level not differing from that what was seen after stimulation with TGF-β_1 _alone was shown at both protein (A) and mRNA (B) level. There was no statistical difference between the inhibitory effects of MLK and BAY u9773 in any setting. Data are expressed as mean ± SEM (n = 12) *p < 0.05, **p < 0.01, and ***p < 0.001. NS – non-significant.

**Figure 6:**
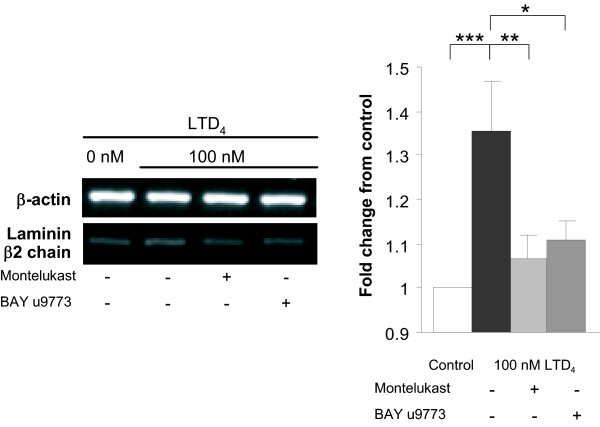
**Inhibition by antileukotrienes of laminin (Ln) β2 chain expression in cysteinyl leukotriene-stimulated BEAS-2B human bronchial epithelial cells**. RT-PCR analysis of Ln β2 chain mRNA showing significant inhibition of the 100 nM LTD_4_-induced up-regulation by both montelukast (MLK) and BAY u9773. There was no statistical difference between the inhibitory effects of MLK and BAY u9773. Data are expressed as mean ± SEM (n = 12) *p < 0.05, **p < 0.01, and ***p < 0.001.

Both CysLT receptor antagonists montelukast and BAY u9773 abolished the significant CysLT-induced increase in Tn production at both protein (Figure 5A) and mRNA level (Figure 5B). This inhibition was complete as the levels of Tn expression after receptor blockade did not differ from that in non-stimulated cells. Similarly, the significant up-regulation of expression of Ln β2 chain mRNA by 100 nM LTD_4 _was fully prevented by both montelukast and BAY u9773 (Figure 6). There was no statistical difference between the inhibitory effects of the CysLT_1_-specific antagonist montelukast and the dual antagonist BAY u9773 in any setting. In experiments, where LTD_4 _was combined with 1 ng/ml TGF-β, both montelukast and BAY u9773 reduced the expression of Tn protein and mRNA down to the level not statistically differing from that what was seen after stimulation with TGF-β alone (Figures 5A and 5B). Neither of the antileukotrienes alone significantly affected the baseline Tn and Ln β2 chain expression. Neither montelukast nor BAY u9773 significantly affected the TGF-β-induced Tn-expression (Figures 5A and 5B).

## Discussion

In this study, we showed for the first time that CysLTs are able to significantly augment the expression of Tn by human bronchial epithelial cells. However, only LTD_4 _is potent enough to enhance the expression of Ln β2 chain. We also demonstrated that the CysLT-induced increase in Tn and Ln β2 chain is mediated solely by the CysLT_1 _receptor, as montelukast, a specific CysLT_1 _inhibitor, completely abolished this increase and dual inhibition of both CysLT receptors did not provide any significant additional effect.

It is remarkable that the CysLTs can produce increased expression of Tn and Ln β2 chain even alone suggesting that they act as independent remodeling-inducing mediators at least on human bronchial epithelial cells. This is apart from that shown in a former study on human lung fibroblasts in which LTD_4 _only augmented TGF-β-induced collagen production, but had no effect alone [12]. One could argue, however, for that the increased collagen leading to fibrosis on the one hand and Tn along with Ln β2 chain on the other may convey somewhat different significance with regard to tissue remodeling. Indeed, in contrast to collagens, Tn is an ECM protein, which shows rapid tissue turnover represented by prompt increase followed by a decrease either after discontinuation of the adverse situation [10] or as a result of treatment [7]. However, increased Tn has been suggested to have dual significance within the airways. Beyond the role of acutely up-regulated Tn in the inflammatory response and cell trafficking, the deposition of Tn into the airway mucosa provides an addition to the increased accumulation of the ECM components, such as collagens, leading to increased airway narrowing and reduction in airway wall compliance [10]. The accumulation of collagens and Tn share even more common aspects. Tn is a matricellular protein that facilitates both morphogenesis of epithelial organs and re-epithelization after injury, but is also responsible for correct rebuilding of the ECM, including proper deposition of collagen [27]. This pertains with minor reservations also to the Ln β2 chain, which is transiently expressed at the epithelial-mesenchymal border and mediates morphogenetic events and organization of the basement membrane (BM) up to the canalicular stage of lung development [28]. In addition, increased expression of both Tn [7] and Ln β2 chain [5] have been shown in the subepithelial BM in patients with asthma in a manner paralleling the severity and duration of the disease. Not only this refers to the involvement of these proteins in the remodeling of the ECM, but suggests also a reverse quantitative expression pattern, compared to that observed during the human fetal airway morphogenesis. In this light, the increased synthesis of Tn and Ln β2 chain by bronchial epithelial cells in response to CysLTs in the present study may be reminiscent of an inflammatory response in which CysLT-rich milieu is a characteristic feature, like this is the case in asthma [29]. It has been suggested that inflammatory mediator-induced submucosal remodeling is initiated through the bronchial epithelium [30]. According to this widely accepted model, the stimulated or injured epithelial cells drive the mesenchymal cells within the epithelial-mesenchymal trophic unit in a way similar to the processes during the fetal development [3]. Our present findings supplement this knowledge with the ability of the bronchial epithelial cells to contribute to the airway remodeling even independently on the communication with mesenchymal cells.

To strengthen the evidence that CysLTs act directly on the human bronchial epithelial cells that results in increased Tn and Ln β2 chain synthesis, we next focused on the expression of the two CysLT receptors on these cells. Of the overall human respiratory epithelium, the presence of CysLT_1 _and CysLT_2 _have been formerly described immunohistochemically on nasal mucosal epithelium in both healthy individuals and patients with chronic rhinosinusitis [31]. Both CysLT receptors have been described also in the epithelium of palatine tonsils of pediatric patients with sleep apnea and recurrent throat infections [32]. Recently, with use of immunohistochemistry and *in situ *hybridization, a weak and moderate expression of CysLT_1 _has been described in bronchial epithelial cells of controls and asthmatics, respectively [33]. Interestingly, only some but not all bronchial epithelial cells appeared to show expression of the CysLT_1 _protein in that particular study and unfortunately, expression of CysLT_2 _was not addressed. Thus, the expression of both CysLT receptors on BEAS-2B human bronchial epithelial cells at both mRNA and protein level in the present study is partly in line with and partly supplement the data obtained by previous investigators. The evidence we have obtained that the CysLT_2 _is more abundant on human bronchial epithelial cells than the CysLT_1 _is supported by the results of an immunohistochemical study of Corrigan and co-authors on nasal epithelial cells [31]. RT-PCR analyses in our present study did support our immunofluorescence and flow cytometry findings strengthening the possibility that the stronger immunoreactivity for CysLT_2 _reflects the true higher expression of CysLT_2 _compared to that of CysCT_1 _rather than results from different antibody performances. Our Western blot analysis of the CysLT_1 _displayed several protein bands, which obviously correspond to the oligomeric forms (Figure 4A). This is in agreement with previous reports, which along with the monomeric form have demonstrated larger amounts of dimerized and oligomerized CysLT_1 _even in the presence of denaturing gel detergents [34]. When probing for CysLT_2_, also several specific immunoreactive bands were detected with a major band at approximately 148 kDa, corresponding most likely to the oligomers of the receptor. Although the evidence that the CysLT receptor immunoreactivity was completely blocked by the blocking peptides supports the specificity of the detected bands, the molecular weights do not precisely correspond to monomers and homo-oligomers of the CysLT receptors. This could be explained by post-translational modifications, but rather by the possibility of composing heterodimers and hetero-oligomers, which is a characteristic feature of G-protein-coupled receptors [35].

The CysLT-induced increase in Tn and Ln β2 chain production by bronchial epithelial cells was completely abolished by both CysLT_1_-specific antagonist montelukast and a dual antagonist for both CysLT_1 _and CysLT_2 _receptors, BAY u9773. Furthermore, increased Tn expression as a result of combined stimulation with LTD_4 _and 1 ng/ml TGF-β was reduced by both montelukast and BAY u9773 down to the levels not statistically differing from that what was seen after stimulation with TGF-β alone. This may refer to the possibility that CysLTs may affect Tn expression independently of TGF-β. Since montelukast appeared to be sufficient to block the effect of the CysLTs and there was no difference between the two antileukotrienes for their inhibition activities, the results allow us to suggest that the CysLT-induced increase in Tn and Ln β2 chain in bronchial epithelial cells is mediated through the CysLT_1 _receptor.

## Conclusion

Our present results demonstrate for the first time that the CysLTs LTD_4 _and LTE_4 _augment production of Tn, whereas LTD_4 _enhances also synthesis of Ln β2 chain by human bronchial epithelial cells and this effect is mediated via the CysLT_1 _receptor. The results not only point to the possibility that, when exposed to CysLTs, the epithelial cells can participate in the development of airway remodeling independently on communication within the epithelial-mesenchymal trophic unit, but also implies that treatment with CysLT_1 _receptor antagonists can be effective to suppress this important mechanism of airway remodeling in asthma.

## Competing interests

The authors declare that they have no competing interests.

## Authors' contributions

SA conceived and designed the study, coordinated all stages of the laboratory-based experiments and analyzed the flow cytometry, Western blot, and RT-PCR results. She also contributed significantly in preparation of the manuscript, MK and ER handled the cell work, as well as analysis of the results, as well as were involved in drafting the manuscript, AA elaborated the conception and participated in the design of the study, accomplished the data analysis, supervised the interpretation of the data, and critically evaluated and supplemented the manuscript.
